# Sulfamethoxazole-induced sulfamethoxazole urolithiasis: a case report

**DOI:** 10.1186/s12894-021-00894-5

**Published:** 2021-09-17

**Authors:** Megan M. Roedel, Stephen Y. Nakada, Kristina L. Penniston

**Affiliations:** 1 Wisconsin Academy for Rural Medicine, University of Wisconsin School of Medicine and Public Health, Madison, WI USA; 2 Department of Urology, University of Wisconsin School of Medicine and Public Health, 1685 Highland Avenue, 3258 Medical Foundation Centennial Building, Madison, WI 53705-2281 USA; 3 Department of Radiology, University of Wisconsin School of Medicine and Public Health, Madison, WI USA

**Keywords:** Drug-induced urolithiasis, Trimethoprim-sulfamethoxazole, Nocardia, Case report

## Abstract

**Background:**

Drug-induced urolithiasis falls into two categories: drug-induced and metabolically-induced. Certain antimicrobials are associated with each; sulfonamides are associated with drug- or metabolite-containing calculi when taken in large doses over a long period of time. Trimethoprim-sulfamethoxazole, a member of the sulfonamide family, is a rare cause of drug-induced calculi. Cases of sulfonamide urolithiasis occurring in patients with known stone disease have rarely been reported.

**Case presentation:**

We report a case of a patient with a brief history of recurrent calcium oxalate nephrolithiasis requiring 2 ureteroscopic procedures whose existing 6 mm lower pole renal stone more than quadrupled in size to form a 4 cm renal staghorn after 4 months of high-dose treatment for *Nocardia* pneumonia with trimethoprim-sulfamethoxazole. After ureteroscopy with laser lithotripsy and basketing of fragments, the stone was found to be predominantly composed of N^4^-acetyl-sulfamethoxazole, a metabolite of sulfamethoxazole.

**Conclusion:**

Stones composed of sulfamethoxazole or its metabolites are rare but have known associated risk factors that should be considered when prescribing this antibiotic. This case report illustrates additional risk factors for consideration, including pre-existing urinary calculi that may serve as a nidus for sulfamethoxazole deposition, and reviews treatment and prevention methods.

## Background

Drug-induced kidney stones account for up to 2% of all urinary tract calculi [1]. Typically, these drugs fall into one of two categories: (1) drugs that have high renal excretion and are poorly soluble in urine; and (2) drugs that induce metabolic changes that favor urinary stone formation. Drugs in the first category, or metabolites thereof, are identified as components of calculi whereas drugs in the second category lead to formation of calcium- and/or uric acid-containing stones. Select drugs within the drug-containing calculi category, including the antihypertensive diuretic triamterene and the nonsteroidal analgesic glafenine, are now used less frequently due to their lithogenic potential; others continue to be used due to their treatment efficacy—these include indinavir and other protease inhibitors used in the treatment of HIV [1]. Metabolically-induced calculi containing calcium can be caused by hypercalciuria from excessive calcium and vitamin D supplementation. Other metabolism-altering drugs, such as carbonic anhydrase inhibitors (e.g., acetazolamide, topiramate) contribute to calcium oxalate and calcium phosphate stone formation by dramatically lowering urinary citrate excretion. Because they significantly raise urine pH, the risk for calcium phosphate stones in particular is increased. Uric acid-containing metabolic calculi can be induced by laxative abuse or by uricosuric agents such as benzbromarone used to treat gout [1].

Several antimicrobial agents are associated with urinary tract calculi [1, 2]. Some act by altering the gut microbiome and its ability to degrade oxalate [3], leading to the formation of hyperoxaluria and calcium oxalate stones. Others, especially when taken in large doses over a long period of time, are known to form urinary crystals and calculi that contain the drug itself or its metabolite(s); these include ciprofloxacin, ceftriaxone, and sulfonamides [1]. Trimethoprim-sulfamethoxazole (TMP-SMX) is a combination antibiotic of the sulfonamide family used to treat a wide variety of commonly encountered infections, including those affecting the urinary tract, respiratory system, and gastrointestinal tract. It is also indicated in the treatment and prophylaxis of opportunistic infections by *Pneumocystis carinii* and *Nocardia spp* [4]. While first-generation sulfonamides were well-known to provoke drug-induced urolithiasis, it is significantly less common with newer sulfonamides and even less common with TMP-SMX [1]. Cases of sulfonamide urolithiasis occurring in patients with known stone disease have rarely been reported.

Here we present a case of a patient with a known kidney stone who was subsequently treated with a long course of high-dose TMP-SMX for *Nocardia* pneumonia. After 4 months of TMP-SMX therapy, the patient’s known renal stone more than quadrupled in size to form a 4 cm staghorn stone with an accompanying obstructive conglomerate of ureteral stones. From fragments obtained during surgical stone removal, composition was predominantly N^4^-acetyl-sulfamethoxazole.

## Case presentation

The patient was a 64-year-old woman with gastroesophageal reflux disease, asthma (treated with inhaled but not systemic steroids), obesity, and type II diabetes mellitus (treated with metformin extended release 500 mg and glipizide 5 mg twice daily). Her stone onset was characterized by presentation to emergency care for nausea, vomiting, diarrhea, and lower left quadrant abdominal pain. While awaiting computed tomography (CT), she acutely became febrile and tachycardic. Labs were significant for white blood cell count of 20,000 K/uL, lactate of 2.2 mmol/L, creatinine of 1.98 mg/dL (baseline 1.06), and concurrent infection on urinalysis. CT scan revealed a 1 cm obstructive proximal ureteral stone. She was diagnosed with sepsis and acute kidney injury and was admitted for decompression and antibiotic therapy; she was discharged after 3 days. Immediate left ureteroscopy (URS) was scheduled but then postponed due to the COVID-19 pandemic. Ultimately, 2 months post presentation, she underwent left URS with laser lithotripsy (LL). Stone composition was unknown. A 24-h urine collection demonstrated high oxalate and low citrate; other risk parameters were within normal limits. She initiated medical management in our multidisciplinary stone prevention clinic for these risk factors. Further assessment revealed positive family history for nephrolithiasis (sister), history of prior ascorbic acid supplementation, very low calcium intake, and high suspicion for antibiotic-induced dysbiosis. The regimen for reducing urine oxalate was to: (1) not re-start ascorbic acid supplement to protect against higher oxalate biosynthesis; (2) pair calcium-containing foods or beverages with every meal to reduce bioavailability of dietary oxalate and, hence, its urinary excretion; and (3) increase intake of prebiotics from fruits and vegetables in effort to enhance colonization and proliferation of oxalate-degrading probiotics (bacteria) in the digestive tract. The regimen for raising urine citrate was to increase intake of fruits and vegetables because they provide bicarbonate precursors that reduce renal citrate reabsorption and thus increase its excretion. Three months later she was found in follow-up to have an 8 mm left renal calculus which was removed within 2 weeks by URS with LL.

**Fig. 1:**
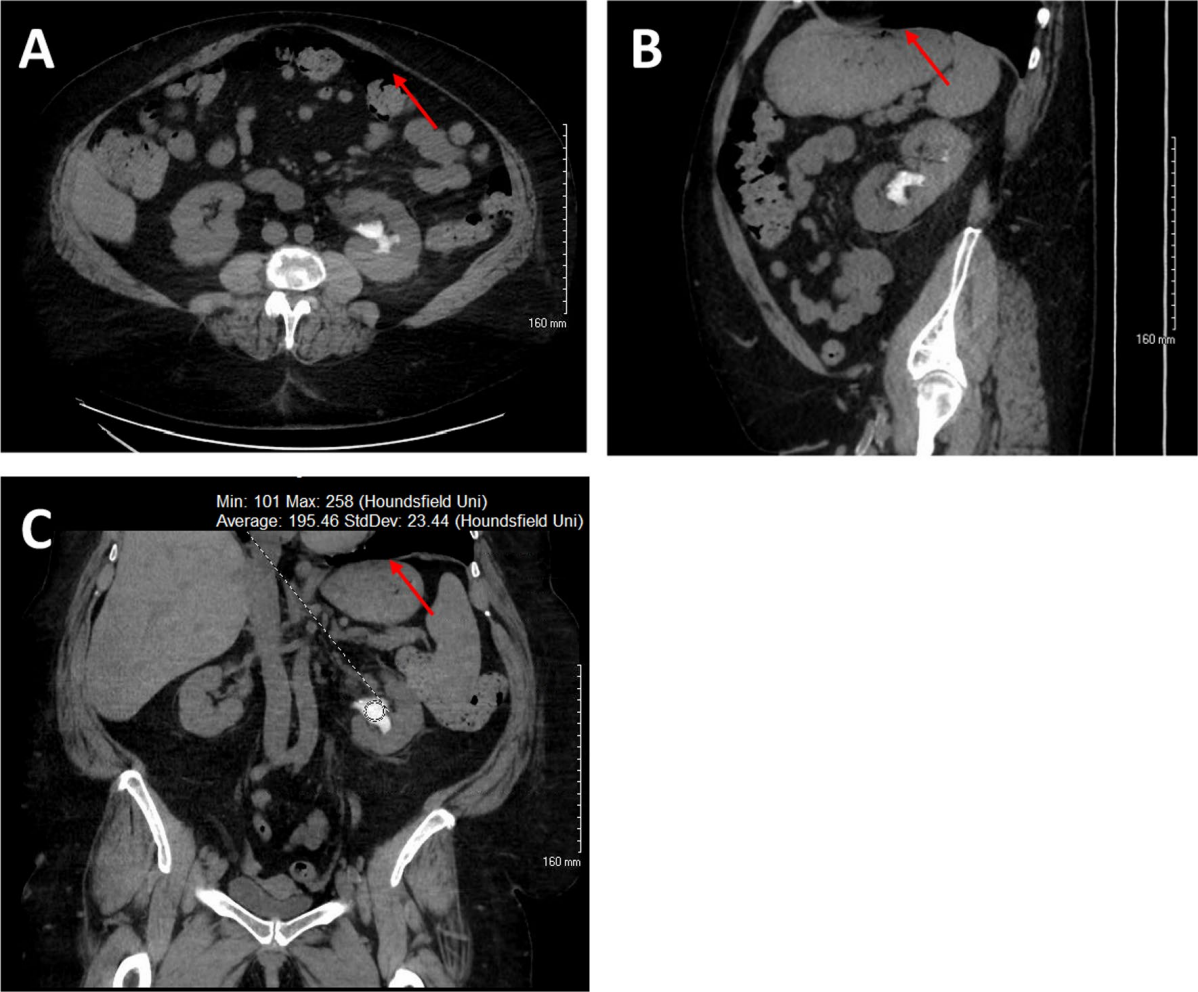
Radiographic images of left renal pelvis stone (red arrow) in the axial (**a**), sagittal (**b**), and coronal (**c**) on non-contrast CT abdomen and pelvis. The stone was demonstrated on imaging to have a density of 194.46 Hounsfield Units

**Fig. 2:**
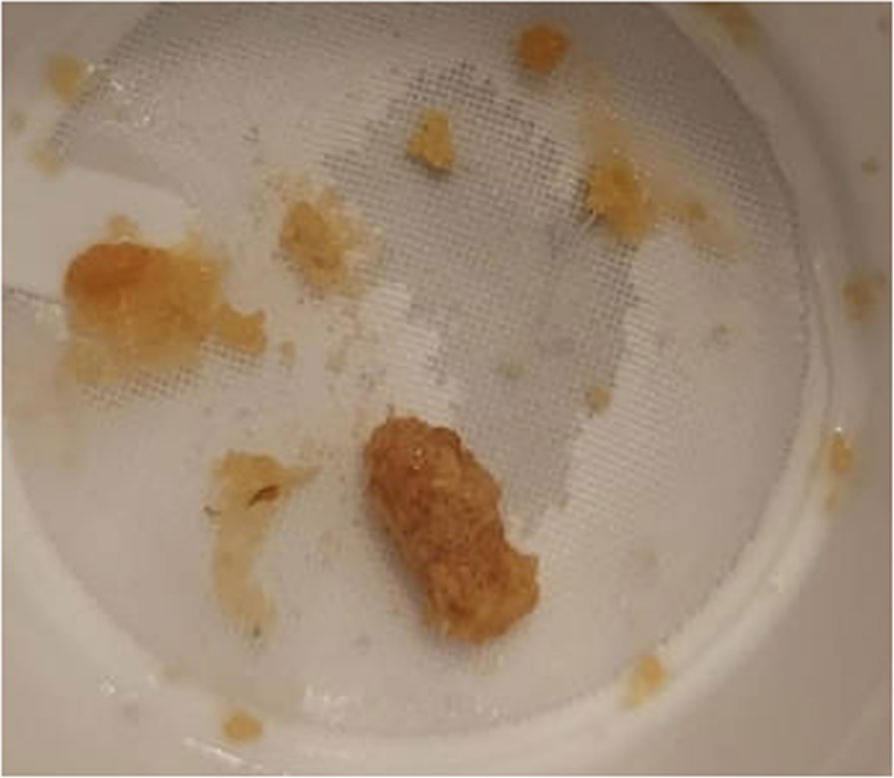
Jagged, amber-colored stone fragments passed and collected by patient after ureteroscopy

Shortly thereafter she was evaluated by infectious disease for a worsening productive cough over several months and was found to have *Nocardia* pneumonia with pulmonary nodules seen on CT scan. She was subsequently started on a six-month course of twice daily TMP-SMX 2DS and cefpodoxime 200 mg. Prior to starting the antibiotic regimen, a non-contrast CT scan showed a new 6 mm non-obstructing stone in the left lower renal pole. A repeat CT scan three months later (2 months after starting TMP-SMX) revealed interval growth of the renal stone to 9 mm; intervention with URS was scheduled. But in the interim, the patient became symptomatic and presented for emergency treatment. Physical exam was significant for mild pelvic tenderness. Labs were significant for creatinine of 1.58 mg/dL and hematuria but no infection. Her pain was managed, and she was discharged. Repeat CT, now 4 months after starting TMP-SMX, revealed a developing staghorn calculus in the left renal pelvis measuring up to 4 cm (Fig. 1) and an obstructing conglomerate of calculi in the left lower ureter measuring up to 7 mm with mild upstream hydroureteronephrosis. Within 2 days, the patient underwent left URS and LL of the renal pelvis and ureteral stones. During the procedure, stone fragments were retrieved via basket and sent for analysis; results demonstrated 80% N^4^-acetyl-sulfamethoxazole with remaining constituents calcium oxalate (12% monohydrate and 5% dihydrate) and 3% calcium phosphate carbonate apatite (composition was assessed by Fourier transform infrared spectroscopy at Dianon Systems in Oklahoma City, OK). The origin of the fragments that were analyzed, whether from the larger renal pelvis stone or from the ureteral conglomerate of stones, was not documented and is thus unknown. Afterward, the patient passed multiple amber-colored stone fragments (Fig. 2).

The patient completed 6 months of antibiotics with resolution of lung nodules seen on CT and improvement of respiratory status to baseline. TMP-SMX and cefpodoxime were thus discontinued. A repeat CT a few months later showed clearance of post-URS fragments and no new urolithiasis bilaterally. She continues to follow in our stone prevention clinic.

## Discussion and conclusions

Sulfamethoxazole is a member of the sulfonamide family, a group of short-acting antibiotics given via orally or parenterally. They are readily absorbed systemically, partially acetylated in the liver, and eliminated by the kidney [4, 5]. The first generation sulfonamides developed in the late 1930s had low urine solubility and were known to cause drug-induced crystalluria resulting in acute renal injury or obstructive urolithiasis [5]. Sulfonamides with higher urine solubility have since been developed, and sulfonamide crystalluria has become a rare occurrence. According to a study by Albala et al., sulfonamide stones make up less than 1% of all stones analyzed [2].

Sulfamethoxazole and its metabolite, N^4^-acetyl-sulfamethoxazole, have particularly low lithogenic potential due to the losangic shape of the crystals, which are not readily retained in the kidney [1]. There have been few reports of N^4^-acetyl-sulfamethoxazole-induced urolithiasis over time except in recent years, during which TMP-SMX has been used for long-term prophylactic treatment of HIV-associated infections [6]. According to Barnes and Kawaichi, the most important factors in urinary precipitation of sulfonamides include concentration of the drug in the urine, degree of acetylation, urinary stasis, urine pH, and urine temperature [7]. The patient in this case was undergoing prolonged treatment with TMP-SMX at high doses for *Nocardia* pneumonia, which likely led to a high urine drug concentration. Her history of type II diabetes mellitus and the associated insulin resistance may have contributed to a more acidic urine pH at baseline [8]. She also had a change in urine pH from 6.5 prior to beginning the antibiotics to 5.5 at the time of symptomatic presentation. In addition, the pre-antibiotic 6 mm lower left renal stone (which was presumably calcium oxalate) may have acted as a nidus for sulfamethoxazole crystal deposition. It is not clear if this is the case as the stone was submitted for analysis in fragment form with no discernable core.

The patient had a known renal calculus at the time of starting TMP-SMX, which is a relatively rare finding. Albala et al. identified 40 people who developed sulfonamide stones, four of whom had a history of prior urolithiasis; however, none had a known stone at the time of treatment [2]. Siegel described a patient who presented with oliguria while on a 2-week course of TMP-SMX for prostatitis who was found with bilateral ureteral stone obstruction; analysis revealed a pure N^4^-acetyl-sulfamethoxazole stone on one side and a calcium oxalate stone contralaterally [9]. However, unlike our patient, he had no prior history of nephrolithiasis; presence of his stone was unknown until presentation.

The treatment and prevention of sulfonamide crystalluria and urolithiasis has not changed significantly since the 1940s and, given the rarity of cases, no standard regimen is established. The mainstays of treatment include discontinuation of the sulfonamide if possible, diuresis, and urinary alkalization, as sulfonamides have greater solubility at alkaline pH. Prevention techniques include avoiding sulfonamides if alternative treatments are available, encouraging patients to increase fluid intake when taking sulfonamides, and urinary alkalization with sodium bicarbonate or other alkali [1, 2, 5]. The presence of known renal stones or other foreign bodies such as indwelling catheters should be considered when selecting a sulfonamide for long-term use, as these may be reasons to avoid this family of antibiotics in such patients.

Stones composed of sulfamethoxazole or its metabolites are rare as they do not readily precipitate in urine. Factors such as high drug concentration in urine, urinary stasis, and low urine pH increase this risk. Findings from our report suggest that additional risk factors are prior stone history, pre-existing metabolic (urinary) risk factors, and pre-existing urinary calculi that may serve as a nidus for sulfamethoxazole deposition. Treatment and prevention include diuresis, urinary alkalization, treatment of underlying metabolic and urinary stone risk factors, and use of an alternative antibiotic if possible.

## Data Availability

Data sharing is not applicable to this article as no datasets were generated or analyzed during the current study.

## Abbreviations

CT: Computed tomography
HIV: Human immunodeficiency virus
LL: Laser lithotripsy
TMP-SMX: Trimethoprim-sulfamethoxazole
URS: Ureteroscopy
